# Gestational ketogenic diet programs brain structure and susceptibility to depression & anxiety in the adult mouse offspring

**DOI:** 10.1002/brb3.300

**Published:** 2014-12-29

**Authors:** Dafna Sussman, Jurgen Germann, Mark Henkelman

**Affiliations:** 1 Physiology and Experimental Medicine, The Hospital for Sick ChildrenToronto, Ontario, Canada; 2 Mouse Imaging Center (MICe), The Hospital for Sick ChildrenToronto, Ontario, Canada

**Keywords:** Behavior, development, ketogenic diet, magnetic resonance imaging, neuroimaging, prenatal programming

## Abstract

**Introduction:**

The ketogenic diet (KD) has seen an increase in popularity for clinical and non-clinical purposes, leading to rise in concern about the diet's impact on following generations. The KD is known to have a neurological effect, suggesting that exposure to it during prenatal brain development may alter neuro-anatomy. Studies have also indicated that the KD has an anti-depressant effect on the consumer. However, it is unclear whether any neuro-anatomical and/or behavioral changes would occur in the offspring and persist into adulthood.

**Methods:**

To fill this knowledge gap we assessed the brain morphology and behavior of 8-week-old young-adult CD-1 mice, who were exposed to the KD in utero, and were fed only a standard-diet (SD) in postnatal life. Standardized neuro-behavior tests included the Open-Field, Forced-Swim, and Exercise Wheel tests, and were followed by post-mortem Magnetic Resonance Imaging (MRI) to assess brain anatomy.

**Results:**

The adult KD offspring exhibit reduced susceptibility to anxiety and depression, and elevated physical activity level when compared with controls exposed to the SD both in utero and postnatally. Many neuro-anatomical differences exist between the KD offspring and controls, including, for example, a cerebellar volumetric enlargement by 4.8%, a hypothalamic reduction by 1.39%, and a corpus callosum reduction by 4.77%, as computed relative to total brain volume.

**Conclusions:**

These results suggest that prenatal exposure to the KD programs the offspring neuro-anatomy and influences their behavior in adulthood.

## Introduction

The ketogenic diet (KD), a known treatment for intractable epilepsy, has been recently found efficacious in treating and/or managing a variety of other conditions; from type-II diabetes, to Alzheimer's disease and cancer (Veech et al. 2001; Van der Auwera et al. 2005; Zhou et al. 2007; Hussain et al. 2012). This very low-carbohydrate, high-fat diet has also been applied in non-clinical settings, and has been adopted as a lifestyle choice by healthy individuals. The increase in popularity of the KD has elicited concern among clinicians about the diet's impact on following generations. Specifically, if women are adhering to a KD during gestation, would this diet have any implications for their offspring? Would their offspring develop and behave normally? And would they be at an increased risk for developing neurobehavioral disease?

The fact that a KD does reduce frequency of epileptic seizures clearly indicates that the diet has a neurological effect (Bough and Rho 2007; Hartman et al. 2007). In fact, the KD was found to significantly alter the levels of metabolites of serotonin and dopamine, suggesting its mechanism of action may be through these mono-amine neurotransmitters (Dahlin et al. 2012). Since these neurotransmitters play a role in anxiety and depression, the KD may actually confer anti-depressant and anti-anxiety properties, as observed and suggested by Murphy et al. (2004). The KD has also been reported to increase the vascular density in the brain (Puchowicz et al. 2007), and protect against neuronal loss (Maalouf et al. 2009; Jiang et al. 2012), implying the diet can alter the anatomy as well as the chemistry of the brain.

To the best of our knowledge, no study has thoroughly investigated the neuroanatomical along with the behavioral effects of prenatal exposure to a KD. While the above-cited studies did investigate the implications of following a KD, none investigated the implications for the subsequent generation, in the case when the diet is followed during gestation.

In our previous studies we started investigating the physiological consequences of a gestational KD on the offspring in mice (Sussman et al. 2013a,b). Those studies focused on the embryonic and neonatal periods and revealed embryonic growth retardation, and alterations in brain structure just prior to parturition, and also in early postnatal life. The current study uses a new group of mice not previously included in our previous papers. This study focuses on the adult mouse offspring, and assesses their brain morphology, using 3-dimensional (3D), high resolution imaging, as well as measuring their susceptibility to anxiety, depression, and altered physical activity level, using standardized neurobehavioral tests (Rogers et al. 1997). Mice prenatally exposed to a KD are compared to those exposed to a prenatal standard diet (SD). In both cases, all mice are fed the SD postnatally until adulthood.

## Materials and Methods

Six-week old male and female CD-1 mice were weight-matched and arbitrarily assigned to either a control group (SD) or a study group (KD). They were kept under controlled room temperature and a 12-h light-dark cycle with ad lib water and their respective diets. After 30 days on their assigned diet, they were naturally mated by setting up a single male with a single female from the same group. The morning newly-born pups were observed was considered day 0.5 of post-natal life (P0.5). Since lactation in the KD dams leads to fatal ketoacidosis (Sussman et al. 2013b), cessation of milk production was induced via separation of the KD dams from their pups by P2.5. The separated KD pups were adopted by lactating SD-foster dams whose pups were at the same age as that of the KDs’. The control group was treated similarly. This ensured the viability of the KD dams as well as that of the pups. At weaning (P21.5), randomly selected SD and KD pups were perfused and their brains imaged with MRI. The remaining pups were allowed to reach early adulthood (8 weeks of age), at which point they underwent neurobehavioral tests and were perfused thereafter. These perfused adult offspring brains were also imaged with MRI, using the same sequence as that used for the P21.5 brains.

### Diet

Both the Standard Diet (SD) and the Ketogenic Diet (KD) were manufactured by Harlan (Madison, WI) (Harlan 2009). The SD provided 3.1 Kcal/g and consisted of 5% fat, 76.1% carbohydrate, and 18.9% protein wt/wt (Teklad diet no. TD.2918). The KD provided 6.7 Kcal/g and consisted of 67.4% fat, 0.6% carbohydrate, and 15.3% protein wt/wt (Teklad diet no. TD.96355). The KD contained a 4:1 ratio by weight of fat to combined protein and carbohydrate, consistent with the formulation of the classical ketogenic diet. Food and water were supplied ad lib prior to mating, during gestation, and post parturition to all mice, independent of diet.

### Animals

All animal procedures in this study were carried out in accordance with the standards of the Canadian Council on Animal Care, and approved by the Animal Care Committee of the Toronto Centre for Phenogenomics (TCP). Prior to mating, body weight, blood glucose and *β*−ketones (*β*−*hydroxybutyrate*) were measured in randomly chosen animals from both groups. Blood concentrations were measured using an Abbott “Precision Xtra” glucometer, which required a relatively minute blood volume of 0.6–1.5  *μ*L/test (Abbott 2006) drawn from the tail vein.

Adoption of P2.5 KD and SD pups by SD foster dams was conducted by mixing the adopted pups with soiled wet bedding from the foster dam's cage. This ensured adopted and biological pups were similar in scent. Since the litter size of the SD foster dam was to not exceed a 30% increase compared with the biological litter size, these dams could only keep about half of their biological pups. The tails of the biological pups that were kept were clipped, for easy recognition. After adoption, cages were not disturbed for 48 h, and afterwards were only interrupted once a week for cage and/or food changes. Any biological or adopted pups that were not adopted by the SD foster dam, were euthanized.

At P21.5 all pups were weaned, separated by gender and litter, and ear-notched for identification. Of the mice that did not undergo the neurobehavioral tests, a subset was randomly selected to undergo weight, blood glucose, and blood ketone measurements at 8-weeks of age. This subset consisted of 24 adopted SD mice (13 F and 11 M) and 20 KD mice (8 F and 12 M).

### Neurobehavioral tests

Neurobehavioral tests were initiated at 8 weeks of age (P56.5), and were concluded at 12 weeks of age (P90.5). Each mouse was handled for 1–2 min per day for 2–3 days prior to beginning the behavioral tests. The battery of tests consisted of the open-field test (OFT), which tested general locomotor activity and anxiety, followed by the forced-swim test (FST), which tested behavioral despair, and by the exercise wheel test (EWT), which tested level of physical activity. Both the OFT and FST took place between 8:00 am and 11:00 am. Since anxiety- and depression-like behaviors are most noticeable during the latter half of each test, only the last 3 min of the OFT and FST were analyzed and reported in the results.

Mice that completed any given test, but whose computerized tracking was inaccurate or failed to start on time, were excluded from the data analysis. Only mice that completed all tests and whose data were included in all data analyses are reported in this paper. Some SD mice that completed all tests were randomly excluded in order to maintain a roughly 2:1 ratio between KD and SD mice, and the same ratio of males to females in the KD compared with SD groups. Overall, 110 KD mice (51 F and 59 M) and 55 adopted SD mice (24 F and 21 M) completed all three tests and were included in the data analyses. The statistical analyses utilized Analysis of Variance (ANOVA) (Girden 1992) with prenatal diet and gender as factors. In cases where gender was not statistically significant, sexes were combined and these combined results were shown.

### Open-field test (OFT)

The open-field consisted of a square arena having dimensions 50  ×  50  cm, which was surrounded by opaque walls having a height of 50 cm, and illuminated by bright light (200 lux). Each mouse was placed at the center of the arena and was given 6 min to explore it freely. A video-camera was positioned above the arena and recorded the mouse behavior. This behavior was then analyzed by a specialized software (EthoVision 8) which computed the distance traveled, the time spent in the central (40  ×  40 cm) versus peripheral regions, and the average speed of motion. The apparatus was sanitized with 1:3:1 Clidox after each mouse (Gerlai et al. 1996).

### Forced-swim test (FST)

The FST was conducted 1.5 weeks after the OFT. Each mouse was placed for 6 min in a clear 5 L Pyrex cylinder measuring 27 cm in height and 17 cm in inner diameter, which was filled to a height of 17 cm (3.5 L). The water was maintained at 25–27
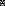
C. The cylinder was sanitized with 70% Ethanol and the water changed between cages. The time spent struggling, actively floating, and immobile were measured in real-time throughout each trial. Struggling was defined as an escape-oriented behavior in which the mouse was moving its limbs rapidly, facing the Pyrex wall, and/or attempting to get out of the water. Actively floating was characterized by motion along the wall of the cylinder or across the center, and involved movement of 3 or 4 limbs. Immobility was defined as lack of motion, or the occasional movement of 1 or 2 limbs that was sufficient only to keep the mouse floating (Petit-Demouliere et al. 2004).

### Exercise wheel test (EWT)

The EWT was conducted 2 weeks after the FST. Each mouse was individually placed in a cage with an exercise wheel for a period of 24 h. An odometer attached to the wheel counted the number of rotations each mouse completed during the test. During the test, mice were provided with ad lib supply of food and water. At the end of each test, the number of rotations was recorded and the cage and wheel were disinfected with Virox Wipes (Virox Technologies Inc., Oakville, ON) (Knab et al. 2009).

### Perfusions

Perfusions took place at two time-points: at weaning (P21.5), and in adulthood (P90.5). The latter perfusion time-point corresponded to the same day - and within 8 h - of completion of the EWT. Mice were initially sedated by an intraperitoneal injection (0.1 mL/10 g of body weight) containing Ketamine and Xylazine, at concentrations of 150 mg/kg and 10 mg/kg, respectively. Mice were then trans-cardially perfused in two steps: (1) Heparin flush – 30 mL of 0.1 mol/L PBS containing 1 *μ*L/mL Heparin and 2 mmol/L Gadolinium (Gd; “ProHance” gadoteridol by Bracco Diagnostics), followed by (2) fixation flush – 30 mL of 0.1 mol/L PBS containing 4% Para-formaldehyde (PFA) and 2 mmol/L Gd. Both perfusion steps were conducted with a fluid flow rate of 100 mL/h. The perfused brain was maintained within the skull; the skin, lower jaw, and eyes were removed. The brain along with remaining skull structure were immersed in 4% PFA containing 2 mmol/L Gd overnight at 4
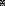
C, and then transferred to 0.1 mol/L PBS containing 0.02% sodium azide with 2  mmol/L Gd for at least 3 days prior to imaging (Nieman et al. 2007; Cahill 2012).

At P21.5 24 adopted SD and 26 adopted KD mice were arbitrarily selected, perfused, and imaged. In adulthood, of the 110 KD mice that completed the neurobehavioral tests, a subset of 82 (41 F and 41 M ) was arbitrarily selected to undergo perfusion and imaging. Seventy-four adopted SD mice (31 F and 43 M) underwent perfusion and imaging. Gender was used as a covariate in the analysis of the adult brain, but was not considered an important factor at the younger timepoint due to it being prior to puberty.

### Brain imaging

Anatomical brain images of the 12-week-old mice were acquired with a 7.0 Tesla Magnetic Resonance Imaging (MRI) scanner (Varian Inc., Palo Alto, CA), using a T2-weighted 3D fast spin-echo sequence. This sequence, which is routinely used for adult mice (Cahill 2012), consisted of an echo train length of 6, TR =  2000 msec, TEeff = 42 msec, field-of-view (FOV) of 25 ×  28  ×  14 mm and matrix size = 450  ×  504  ×  250. This sequence provided an image with 56 *μ*m isotropic voxels. Sixteen brains were imaged concurrently, using a custom-built 16-coil solenoid array (Dazai et al. 2011; Ellegood et al. 2012). In the first phase-encode dimension, consecutive k-space lines were assigned to alternating echoes to move discontinuity-related ghosting artifacts to the edges of the FOV (Thomas et al. 2004). This scheme necessitates oversampling by a factor of 2 in the phase-encode direction to avoid interference of the ghosts with the main image, yielding a FOV of 28 mm that was subsequently cropped to 14 mm after reconstruction. Total imaging time was 11.7 h.

### Image statistical analysis

The image registration process has been previously outlined in detail by Nieman et al. (2007); Sussman et al. (2013a, b). Briefly, all brains were linearly and, subsequently, non-linearly registered together in a common 3D space. For each brain image, a deformation field was created, which transformed each individual image to the common registered space. The determinant of the Jacobian of each deformation field was then computed for each image voxel (3D pixel). This measure revealed local changes in each image with respect to the consensus average image. A statistical analysis of these Jacobians was carried out to determine whether the KD brains significantly differed from the SD (control) ones in any particular 3D region. The result from this analysis is reported as a structural brain image overlaid with a t-statistics map which colors significantly-different voxels in red and blue, as indicated by the accompanying scale-bars. To account for multiple comparisons in this analyses, a False Discovery Rate (FDR) method was utilized (Benjamini and Hochberg 1995; Benjamini 2010), and is reported along with the results. Entire structures were also segmented from the final average image using a pre-existing brain atlas (Dorr et al. 2008); their relative volumes were computed and compared between the two dietary groups. Only regions that were significantly different with an FDR ≤ 5% in this region-based analysis are reported in tabulated form. The results are provided in the form of mean ± standard deviation (Stdv).

## Results

### Neurobehavior in adult offspring

**Figure 1:**
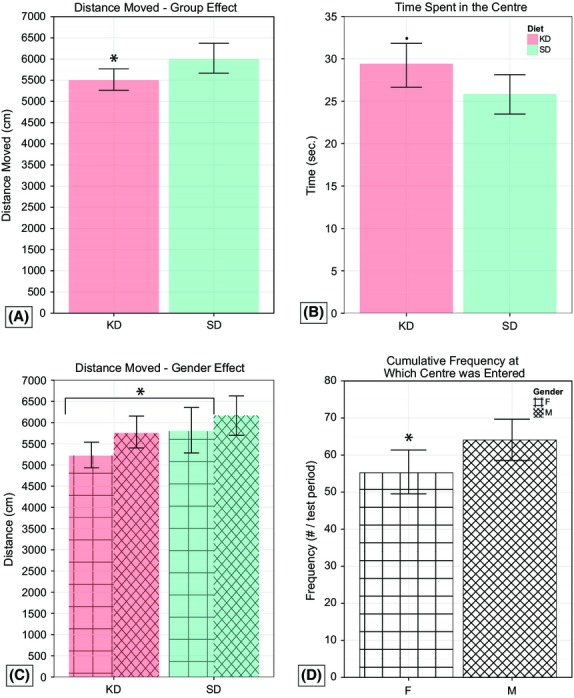
Behavior during the last 3 min of the OFT as a function of diet: (A) Distance travelled - Group effect, (B) Time spent in the center of the arena - group effect, (C) Distance travelled - gender effect, and (D) Cumulative frequency of center visits over the last half of the test - gender effect (Mean ± Stdv; *P* = 0.08; *P ≤ 0.05).

There were notable behavioral differences between the adult adopted offspring, which were prenatally exposed to a KD, and those prenatally exposed to a SD. In the OFT, KD mice, regardless of gender, travelled shorter distances over the last 3 min of the test (Fig.1A). This was accompanied by a slight yet insignificant increase (*P* = 0.08) in time spent in the center of the open-field arena (Fig.1B). Neither gender nor diet affected the distance travelled in the open-field arena (Fig.1C). However, gender did affect the frequency at which mice visited the centre. As shown in Fig.1D, female mice visited the center less frequently than male mice, regardless of their prenatal diet. Hence, not only prenatal diet, but also gender dictates behavior of the adult offspring.

**Figure 2:**
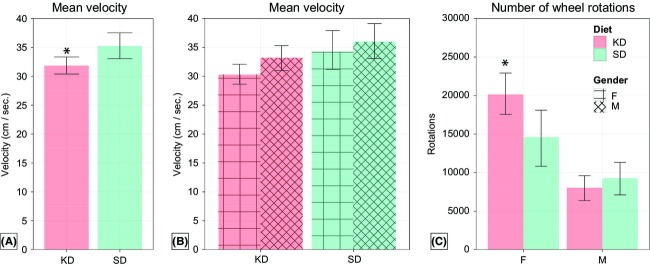
Mean velocity of KD and SD mice in the last 3 min of the OFT: (A) Group effect, (B) Group and Gender effects (insignificant), and (C) Number of wheel rotations in the 24-h EWT *P ≤ 0.05.

The mean velocity during the open-field exploratory motion was also found to be slower for the KD mice, independent of their gender (Fig.2A and B). Assessment of their overall physical activity, as measured by the number of wheel rotations, indicated that the KD females were more physically active than the SD females (Fig.2C).

**Figure 3:**
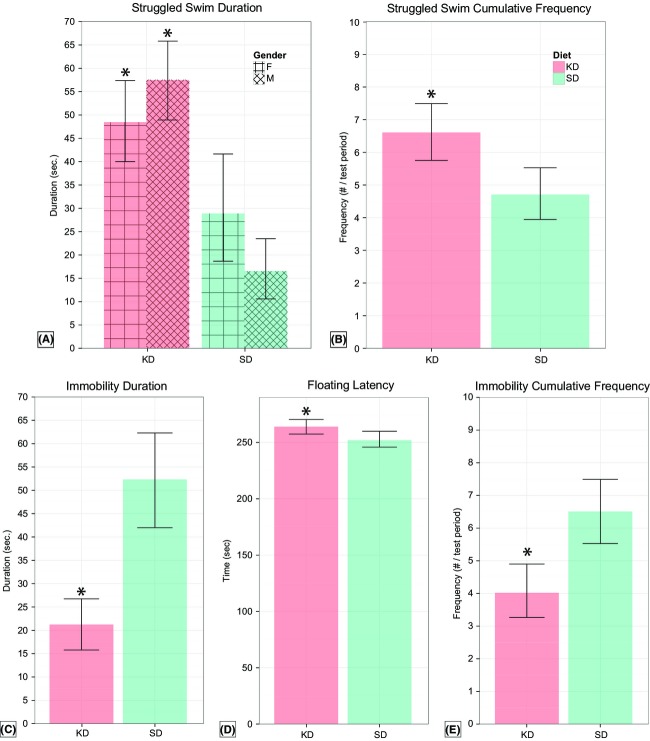
Behavior during the last 3 min of the FST: (A) Struggling duration - Group and Gender effects, (B) Struggling cumulative frequency, (C) Immobility duration, (D) Floating latency, and (E) Immobility cumulative frequency. (Mean ± Stdv; *P ≤ 0.05).

The Forced Swim Test (FST) revealed that the KD mice spent a significantly longer period of time struggling (Fig.3A) and less time immobile (Fig.3C) compared with the SD mice. A similar trend was observed for the frequency of these behaviors, as depicted in Fig.3B and E. That is, the frequency of the struggling behavior was significantly higher, and that of the immobile behavior was significantly smaller, for the KD mice as compared with the SD ones.

There was also an interaction effect of diet and gender: while the SD females struggled significantly more than SD males, the KD females had a tendency to struggle less than KD males, but this tendency did not reach statistical significance.

Analysis of the body weight, blood glucose and blood ketone concentrations of the adult offspring revealed that the male KD mice had a significantly lower blood glucose ( *P*≪ 0.05) compared with the male SD control. A similar trend was seen for the female KD mice, but this difference did not reach statistical significance (*P* = 0.06). Ketone concentration and body weight did not differ between the two dietary groups. These results are summarized in Table 1, along with absolute brain volume, for reference.

**Table 1:** Body weight, blood glucose, blood ketones, and brain volume for adult male and female mice (Mean ± Stdv)

	SD Females	SD Males	KD Females	KD Males
Body weight (g)	29.2 ± 2.9	37.3 ± 3.4	27.9 ± 3.2	36.5 ± 4.4
Blood glucose (mmol/L)	5.9 ± 1.3	7.4 ± 1.0	4.8 ± 1.1	5.1 ± 1.0
Blood ketones (mmol/L)	0.3 ± 0.1	0.4 ± 0.1	0.4 ± 0.2	0.4 ± 0.1
Brain volume (mm 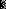 )	527.6 ± 60.5		511.5 ± 54.5	

SD, Standard diet; KD, ketogenic diet.

### Brain structure at weaning and in adulthood

**Figure 4:**
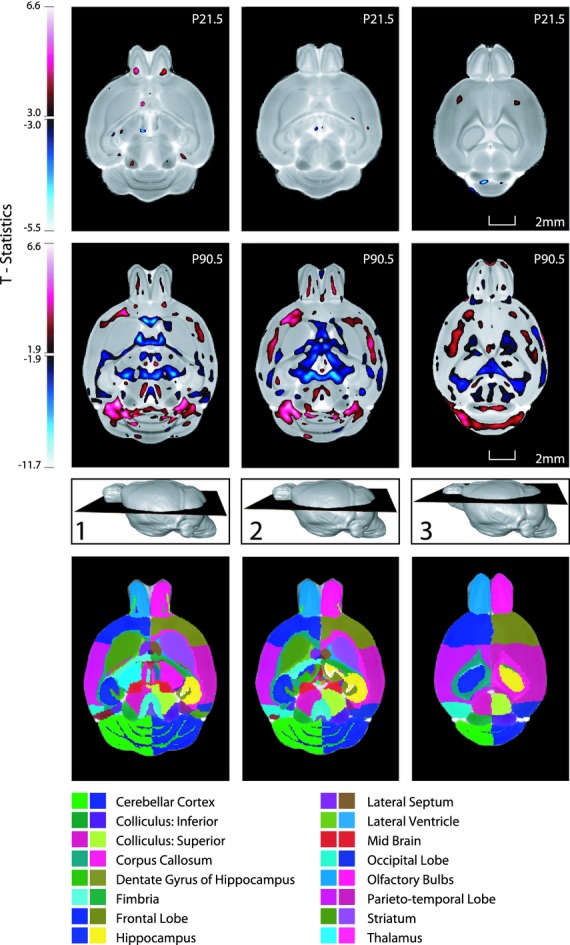
(Top panel) T-statistics map overlaid on top of the registered P21.5 and P90.5 brain images, highlighting voxels with statistically different deformation (FDR ≤ 15%). Blue regions are statistically smaller, whereas red regions are statistically larger in relative volume in the KD compared with the SD brain. Shown are three cross-sectional views of the average P21.5 brains, along with the corresponding cross-sections and (bottom panel) color-coded atlas segmentation at P90.5, for comparison.

Assessment of brain morphology, as conducted by MR imaging and image registration, revealed that the whole-brain volume did not differ significantly with diet either at weaning (SD: 474 ± 43 mm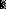, KD: 461 ± 39 mm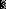) or in adulthood (SD: 528 ± 61 mm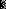, KD: 512 ± 55 mm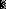), independently of gender (*P* > 0.05). However, several brain structures differed in volume relative to total brain volume in the average KD brain, when compared with the SD one, at both weaning as well as in adulthood. The regional differences in relative brain volume are shown in selected cross-sectional images in Fig.4, where the highlighted regions are those that are statistically different. These images show that the average KD brain had a bilateral increase in relative volume in the frontal cortex, cerebellum, and primary somatosensory cortex, and a bilateral decrease in relative volume in the hippocampus, striatum, motor cortex, and auditory cortex. Additionally, the thalamus and dentate gyrus had a mix of relatively enlarged and relatively smaller regions in the KD brain. As can be seen in Fig.4, most of the relative volume differences are visible in the adult brain, while only a few show up in the younger brain. A region-based analysis was also carried out and the results for all statistically different regions are reported in Table 2.

**Table 2:** Adult brain structures whose relative volumes are significantly different with an FDR  ≤ 5%. Relative volume is provided in the form of Mean ± Stdv

Structure	Relative volume of KD	Relative volume of SD	% Change of KD	*P* value
Anterior commissure: pars posterior	0.086 ± 0.006	0.088 ± 0.005	−2.53	0.01334
Bed nucleus of stria terminalis	0.305 ± 0.018	0.319 ± 0.020	−4.30	<0.0001
Cerebellar cortex	12.057 ± 0.7451	11.505 ± 0.959	4.80	<0.0001
Cerebellar peduncle: superior	0.228 ± 0.012	0.236 ± 0.018	−3.26	<0.01
Cerebral aqueduct	0.146 ± 0.018	0.159 ± 0.040	−7.97	0.01042
Cerebral cortex: entorhinal cortex	2.158 ± 0.080	2.200 ± 0.111	−1.90	<0.01
Cerebral peduncle	0.494 ± 0.019	0.505 ± 0.026	−2.23	<0.01
Colliculus: superior	1.985 ± 0.089	2.036 ± 0.143	−2.49	<0.01
Corpus callosum	3.289 ± 0.140	3.454 ± 0.158	−4.77	<0.0001
Dentate gyrus of hippocampus	0.764 ± 0.043	0.789 ± 0.049	−3.18	<0.001
Fasciculus retroflexus	0.052 ± 0.002	0.053 ± 0.003	−2.07	<0.01
Fimbria	0.689 ± 0.038	0.724 ± 0.055	−4.88	<0.0001
Fornix	0.141 ± 0.006	0.145 ± 0.006	−2.61	<0.0001
Globus pallidus	0.610 ± 0.022	0.634 ± 0.037	−3.76	<0.0001
Hypothalamus	2.355 ± 0.077	2.388 ± 0.090	−1.39	0.01382
Inferior olivary complex	0.042 ± 0.008	0.038 ± 0.010	9.84	0.01024
Internal capsule	0.547 ± 0.024	0.559 ± 0.030	−2.22	<0.01
Lateral septum	0.716 ± 0.030	0.736 ± 0.038	−2.78	<0.001
Lateral ventricle	0.756 ± 0.076	0.792 ± 0.083	−4.47	<0.01
Mammillary bodies	0.123 ± 0.010	0.118 ± 0.008	4.25	<0.001
Midbrain	3.106 ± 0.091	3.156 ± 0.104	−1.58	<0.01
Optic tract	0.334 ± 0.012	0.356 ± 0.015	−6.03	<0.0001
Pontine nucleus	0.172 ± 0.023	0.164 ± 0.020	5.21	0.01554
Posterior commissure	0.037 ± 0.002	0.039 ± 0.002	−5.23	<0.0001
Stratum granulosum of hippocampus	0.190 ± 0.014	0.200 ± 0.017	−5.26	<0.0001
Stria terminalis	0.171 ± 0.010	0.177 ± 0.010	−3.49	<0.0001
Striatum	5.061 ± 0.218	5.165 ± 0.269	−2.02	<0.01
Third ventricle	0.240 ± 0.014	0.252 ± 0.020	−4.56	<0.0001
Ventral tegmental decussation	0.029 ± 0.001	0.030 ± 0.002	−4.24	<0.0001

SD, Standard diet; KD, ketogenic diet.

The region-based analysis revealed significant changes in relative volume in several other brain regions, including a prominent decrease in the hypothalamus, and an increase in the cerebellar cortex. For reference, these changes are larger than those seen in learning and memory studies (Lerch et al. 2010). A comparison between the results from this region-based analysis (Table 2) and the results from the deformation-based analysis (Fig.4) indicates that brain regions that have localized deformations are not necessarily different in overall relative volume.

## Discussion

Our study reveals that prenatal exposure to a ketogenic diet (KD) alters neurobehavior in adult mice. In the open field test (OFT), the average KD mouse travelled shorter distances. While shorter distances travelled in the OFT can, in general, be triggered by anxiety, the fact that this was accompanied by a slower average speed of motion and a slightly increased duration in the center, actually suggests that KD mice were somewhat less anxious about visiting the center of the arena compared with the standard diet (SD) mice. This slight reduction in anxiety may be attributed to the reduced protein contents of the KD compared with the SD (15.3 vs. 18.9%, respectively). In fact, similar behavior was reported in prenatally protein-malnourished rats (da Silva Hernandes et al. 2005; Francolin-Silva et al. 2006; Mokler et al. 2007; Alamy and Bengelloun 2012). These authors speculated that early-life protein malnutrition alters the developing neurochemical systems that underlie behavioral expression in anxiogenic situations (Alamy and Bengelloun 2012). Specifically, since stress triggers a release of dopamine, alterations in dopaminergic pathways were thought to underlie the dampened stress response. Indeed, it was found that prenatal protein malnutrition decreases extra-cellular dopamine release in the prefrontal cortex, hypothalamus, and hippocampus (Davids et al. 2003; Mokler et al. 2007; Alamy and Bengelloun 2012), which can alter the regulation of stress and reward pathways, as well as associated movement.

In the exercise wheel test (EWT), KD mice were found to be significantly more physically active than SD mice. This result was consistent with that of the forced swim test (FST), in which KD mice spent more time struggling. Together these results suggest that KD mice had an increased tendency towards hyperactivity in adulthood. Such behavior may also be attributed to the lower protein contents in the KD, and subsequent alterations in dopamine release. Since the dopaminergic system is believed to play a role in both regulation of reward and movement, it is possible that any prenatal alteration to it resulted in the hyperactive behavior we are seeing here. Another possible explanation could come from ketones’ improvement of mitochondrial respiration (Maalouf et al. 2009). Since improved mitochondrial respiration enhances ATP production, KD mice are likely to have an increase in overall energy to carry out motor function, which could potentially explain their hyperactivity. This speculation is supported by a study that showed that ketosis enhances swimming endurance capacity of mice (Fushiki et al. 1995). Also, the fact that the male KD mice had a significantly lower blood glucose without a concomitant increase in blood ketone concentration compared with SD males, further supports the hypothesis that their metabolism is indeed different.

Regardless of gender, KD mice also exhibited reduced behavioral despair, as measured by the smaller duration of immobility in the FST. A similar lower susceptibility to depression-like behavior has been observed in mice consuming the KD (Murphy et al. 2004); yet, to the best of our knowledge, this effect has not been previously reported in the KD progeny. This indicates that a prenatal KD confers an antidepressant effect, which continues until adulthood, and is not reversed by the postnatal consumption of a standard diet.

While gender did not affect the physical activity of the SD mice, it did affect that of the KD mice, since the KD females used the exercise wheel significantly more than the KD males. Gender, independent of prenatal diet, also affected the frequency at which the mice visited the center in the OFT. Namely, male mice visited the center of the arena more frequently than female mice, regardless of dietary condition, suggesting that male mice experienced reduced anxiety and/or increased exploratory behavior compared with the female mice. In the FST, there was a gender-by-diet interaction: while the SD females struggled more than SD males, the KD females struggled slightly less than the KD males, yet this did not reach statistical significance. This seemingly opposite trend may be attributed to a gender-dependent effect of the KD diet. The effects of gender on rodent neurobehavior have been previously reported (van Haaren et al. 1990; Lopez-Aumatell et al. 2008; Breu et al. 2012; Kazutaka et al. 2012; Rood et al. 2012); however, the underlying mechanisms remain unknown. Recent attempts to elucidate these mechanisms revealed that female mice have a sparser arginine vasopressin (AVP) innervation compared with males (Rood et al. 2012). Since the peripheral action of AVP is to control homeostasis and stress response, as well as to regulate behavioral state, a decrease in AVG innervation in females could result in their heightened stress response.

Our study also reveals that brain morphology of KD mice is altered slightly at weaning (P21.5), but more noticeably in adulthood (P90.5), after 69 days on a SD. Regardless of gender, the adult KD mice have reduced relative volume in the hippocampus, hypothalamus, corpus callosum, striatum, motor cortex, and auditory cortex, and increased relative volume in the cortex and cerebellum. The thalamus and dentate gyrus in the average KD brain both show regions which are enlarged, and others which are smaller compared with the average SD brain. Such volumetric changes may be attributed to the neuro-protective properties of the ketogenic diet, and its effects on neurogenesis. The KD has been found to decrease reactive oxygen species (ROS) formation (Maalouf et al. 2009), thereby protecting the cell against oxidative stress. Since ketones cross the blood-brain barrier though proton-linked monocarboxylic acid transporters (MCT), they can then also enter neurons (again through MCT or diffusion), be incorporated into their ATP synthesis pathway, and reduce oxidative stress in neuronal cells (Nehlig 2004; Morris 2005; Maalouf et al. 2009). It has also been implicated that the KD has an anti-apoptotic effect; it decreased pro-apoptotic factors in the kainic-acid-induced neuro-degenerative animal model (Noh et al. 2003, 2005a, b), increased the activity of the intra-cellular calcium-binding protein *calbindin*, which protects cells against degeneration (McMahon et al. 1998), protected against dopaminergic cell loss in a mouse model of Parkinson's disease (Tieu et al. 2003), and reduced neuronal loss in animal models of hypoxia, hypoglycemia and focal ischemia (Maalouf et al. 2009). The KD has also been found to enhance glial proliferation in the CA3 region of the hippocampus (Silva et al. 2005), and protected against neuronal loss in hippocampal and para-hippocampal cortices in mouse model of amygdaloid-kindling seizures (Jiang et al. 2012). Since decreased neurogenesis – particularly in the hippocampus – has been implicated in the pathogenesis of anxiety and depression, this protective effect of KD on hippocampal neurogenesis could explain the reduced susceptibility to anxiety and depression exhibited by our adult KD mice.

The low protein contents of the KD may also change the neuroanatomy of the KD mouse. Low protein consumption has been shown to reduce dendritic branching in the dentate gyrus (Cintra et al. 1990). Prenatal protein malnutrition has also induced delayed astrogenesis and abnormal neuronal differentiation, and more specifically, abnormal hippocampal formation, altered time course of dentate gyrus development and the morphology of hippocampal cells (Alamy and Bengelloun 2012).

The KD is also known to alter the levels of several hormones, including insulin, glucagon, IGF-1, leptin, and ghrelin (Ahren et al. 1997; Cheng et al. 2003; Veech 2004). Such hormonal imbalance could also play a role in the neuro-anatomical and behavioral changes reported here.

While the studies we cite above do report an effect of diet on neurogenesis, unless stated otherwise, these effects are those observed in the *first* (parent) generation of animals consuming the respective diet. That is, most of these cited studies do not investigate the effects on *offspring* that were prenatally exposed to the offensive environment. Since the impact on the offspring is modulated by the placenta during gestation, and can be altered by any subsequent postnatal neurogenesis (Levitt 2003; Watson et al. 2006), the final alterations that can be detected in the adult offspring may be different than those seen in the parents. This difference can be the cause of discrepancy between our results, and the results reported in the above-cited studies.

One of the shortcomings of our study is that we assumed gender was not an important factor at the first imaging time-point P21.5, due to it being prior to puberty. However, the impact of maternal KD on offspring pubertal age has not been previously assessed. Should puberty in the KD offspring occur at an earlier age that is closer or prior to P21.5, our assumption of the insignificant sexual dimorphism at P21.5 may in fact be inaccurate. In that case, sex should be added as a covariate to the P21.5 analysis. Further studies on the effect of pubertal age in the offspring should attempt to elucidate this.

Overall, the volumetric alterations to brain morphology do imply that a prenatal KD programs subsequent brain development in a way that modifies neurogenesis in multiple brain regions, some of which may underlie the behavioral alterations exhibited by the adult KD mice.

Our study shows that prenatal exposure to a ketogenic diet results in gross differences in brain anatomy as well as behavioral alterations, which include reduced susceptibility to anxiety and depression, and elevated hyperactivity in the adult mouse offspring. Adult male KD mice were also found to have reduced blood glucose concentration compared with SD males. Since both the ketogenic diet (KD) and the standard diet (SD) mice were fed the standard diet postnatally, the alterations we are seeing can only be due to the different prenatal diet. We, therefore, conclude that a prenatal KD programs the offspring neuroanatomy and metabolism, and dictates their behavior in adulthood. Since the mouse and human share high genetic homology, the effects we are seeing on the mouse offspring could be indicative of similar effects human offspring may exhibit if exposed to a KD prenatally. As such, it is important to educate women about the potential effects of a gestational KD to their offspring. To reveal the exact effects on human offspring, human studies need to be conducted. However, human studies will be far more difficult to analyze, due to the high genetic heterogeneity and the vastly more complex environmental factors within human populations.

While our study is the first to reveal behavioral and morphological changes in the KD offspring, there may still be additional behavioral alterations, such as changes in learning and memory, and altered susceptibility to neuro-degenerative disease in older offspring. Hence, future studies should attempt to elucidate any additional behavioral effects, so as to fully characterize the long-term impact of prenatal exposure to a KD.
